# A Case of Incidental Schwannoma Mimicking Necrotic Metastatic Lymph Node from Bladder Cancer

**DOI:** 10.3390/medicina57070728

**Published:** 2021-07-19

**Authors:** Jeong-Hyouk Choi, Koo-Han Yoo, Dong-Gi Lee, Gyeong-Eun Min, Gou-Young Kim, Tae-Soo Choi

**Affiliations:** 1 Department of Urology, College of Medicine, Kyung Hee University, Seoul 05278, Korea; jhchoi0@khu.ac.kr (J.-H.C.); yookoohan@khu.ac.kr (K.-H.Y.); drpedurology@gmail.com (D.-G.L.); danielmin@khu.ac.kr (G.-E.M.); 2 Department of Pathology, College of Medicine, Kyung Hee University, Seoul 05278, Korea; pathogen@khu.ac.kr

**Keywords:** neurilemmoma, retroperitoneal space, urinary bladder neoplasm

## Abstract

*Background and Objectives:* Retroperitoneal schwannoma is a very rare case of schwannoma which commonly occurs in the other part of the body. However, it is difficult to distinguish schwannoma from other tumors before pathological examination because they do not show specific characteristics on imaging study such as ultrasound, computed tomography (CT), and magnetic resonance image (MRI). *Case summary:* A 60-year-old male showed a retroperitoneal cystic tumor which is found incidentally during evaluation of coexisted bladder tumor. Neurogenic tumor was suspicious for the retroperitoneal tumor through pre-operative imaging study. Finally, a schwannoma was diagnosed by immunohistochemical examination after complete surgical excision laparoscopically. *Conclusion:* As imaging technology is developed, there may be more chances to differentiate schwannoma from other neoplasm. However, still surgical resection and histopathological examination is feasible for diagnosis of schwannoma.

## 1. Introduction

Schwannoma (neurilemmomas) are benign tumors that originate from nerve sheath cells along peripheral nerves [1]. They generally form a tumor that is solitary, well-circumscribed, and encapsulated in appearance [2]. Retroperitoneal schwannoma are extremely rare, comprising only 1% of all schwannoma [3]. They are often found incidentally in solid form but frequently undergo cystic degeneration [2]. Surgical excision of the tumor allows for diagnosis and is associated with effective, long-term therapeutic outcomes [4]. We report a case involving successful laparoscopic resection of a pelvic schwannoma.

## 2. Case History

**Figure 1:**
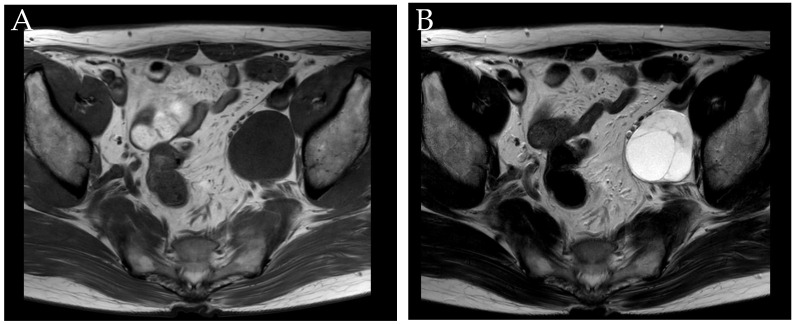
(**A**) A T1-weighted magnetic resonance image shows a homogenous low-signal-intensity mass, while (**B**) the T2-weighted MR image reveals several cysts with septa within the tumor.

A 60-year-old male presented with a two-week history of intermittent gross hematuria. There was a 2.5 cm-sized papillary tumor at the left anterolateral wall of the urinary bladder on cystoscopy. A contrast-enhanced abdominal computed tomography (CT) scan incidentally revealed a 4.8 cm cystic mass with septa at the left pelvic wall. Lymphangioma or cystic changes of the neurogenic tumor were suspicious for a cystic tumor. Magnetic resonance image (MRI) was recommended for differential diagnosis of a necrotic metastatic lymph node, which was the least-likely suspected diagnosis. MRI revealed that the bladder tumor was likely muscle-invasive with Vesical Imaging-Reporting And Data System (VI-RADS) category 4, and the pelvic mass exhibited cystic changes, suggestive of a neurogenic tumor (Figure 1) [5]. There was no evidence of metastases on chest CT and bone scan. The patient was scheduled for transurethral resection of bladder tumor (TURBT) with laparoscopic pelvic mass excision. TURBT alone was performed to confirm the presence of muscle invasion as an initial diagnostic plan.

**Figure 2:**
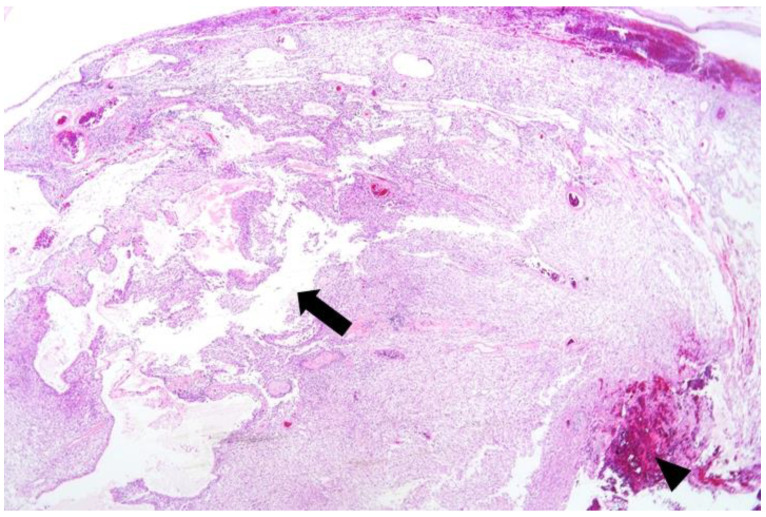
Lower magnification shows a well-defined and capsulated round mass with intratumoral cystic changes (arrow) and hemorrhage (arrowhead) (H-E stain, 20× magnification).

**Figure 3:**
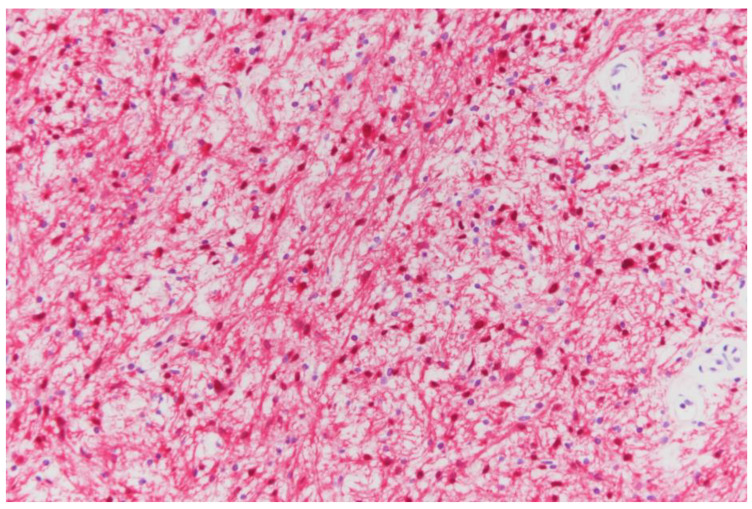
The immunohistochemical study shows diffuse strong-positive signaling for the S-100 protein (200× magnification).

First, TURBT was performed under general anesthesia with the patient in a lithotomy position. The bladder tumor was histologically confirmed as a high-grade papillary urothelial carcinoma, Ta. One month later, laparoscopic pelvic mass excision was completed under general anesthesia using four trocars with the patient placed in the supine position. A 12-mm incision port for the laparoscope plus three other working ports were placed. The left retroperitoneal space was exposed after an incision of the left abdomen peritoneum. The plane was developed by a combination of sharp and blunt maneuvers. The left ureter and left common iliac vessels adherent to the pelvic mass were identified and dissected carefully. Finally, the mass was gently separated from the pelvic wall and extracted into a laparoscopic specimen bag. The specimen was sent to the pathologic department for routine staining and sectioning (Figure 2 and Figure 3). Suction drainage was placed near the resection site. Four days after surgery, he was discharged without any complications. There were no significant lesions on follow-up cystoscopy, or on the abdominal CT taken three months after discharge.

## 3. Discussion

Schwannoma most commonly occur in the head and neck [6]. Although the proportion of retroperitoneal schwannoma is very low, there have been several reports of their occurrence. Schwannoma are usually less than 5 cm in diameter, while those in the retroperitoneal region tend to be much larger [7]. Symptoms are typically absent or nonspecific; however, abdominal pain can manifest as the tumor grows and adjacent structures are compressed [4].

Cystic changes happen when the tumor overgrows its blood supply [8]. Over half of schwannoma appear as cystic lesions on radiography [9]. In this case, a pre-op CT scan showed that the cystic lesion was likely a neurogenic tumor, and the MRI confirmed it to be clear. Although ultrasound, CT, and MRI can visualize the tumor’s features and are helpful; however, they cannot definitively distinguish schwannoma because of the lack of specific features associated with those modalities. Hayasaka et al. reported that benign schwannoma on MR showed low signal intensities on T1-weighted images and high signal intensities on T2-weighted images, in contrast with malignant schwannoma which exhibit mixed-signals and are composed of solid and hemorrhagic cystic components on both T1- and T2-weighted images. However, the authors also mentioned that primary retroperitoneal schwannoma have no specific characteristics [10]. In the literature, only 15.9% of schwannoma were diagnosed correctly using preoperative imaging modalities [11]. Retroperitoneal schwannoma have been misdiagnosed as pancreatic cysts, liver tumors, or psoas abscesses. Even though imaging modalities offer limited characteristics of retroperitoneal mass, CT helps surgeons to understand adjacent structures and select surgical approach, and MRI provides additional tissue analysis including the presence of cystic, necrotic or hemorrhagic component. The difference between this case and other previous reports about the role of imaging may be related to improvements in imaging technology and image reading skill.

Still, surgical excision and immunohistochemical examination are necessary to reach a definitive diagnosis. Intense staining of the S-100 protein is used to confirm the diagnosis of benign nerve sheath tumors, including schwannoma. The recurrence rate is very low in patients with benign schwannoma, while malignant tumors are associated with a very poor prognosis and high recurrence rate [9]. In this case, the schwannoma was detected incidentally during evaluation of a bladder tumor, which was finally confirmed as a localized urothelial carcinoma. We could not determine if the tumor was benign or malignant thorough preoperative examination. Accordingly, we surgically resected the tumor laparoscopically. After immunohistochemical examination, the pelvic tumor was determined to be a schwannoma. No remnant lesion or metastases were found upon completion of a follow-up abdominal CT scan 2 years after the mass resection.

## 4. Conclusions

We reported a case of an incidentally discovered retroperitoneal schwannoma, which is very rare and difficult to diagnose preoperatively. As the use of radiological examinations becomes more common, we expect that there will be increasing cases of incidental schwannoma. However, as imaging studies are limited in their ability to achieve definitive diagnosis, complete surgical resection and regular follow up are necessary.

## Data Availability

The data reported in this paper are available from the medical history of the patient.
